# Sirtuin-mediated nuclear differentiation and programmed degradation in *Tetrahymena*

**DOI:** 10.1186/1471-2121-12-40

**Published:** 2011-09-21

**Authors:** Kristin M Slade, Sydney Freggiaro, Kyle A Cottrell, Joshua J Smith, Emily A Wiley

**Affiliations:** 1 Keck Science Department of Claremont McKenna, Pitzer, and Scripps Colleges, W.M. Keck Science Center, 925 N. Mills Ave., Claremont, CA 91711, USA; 2 Chemistry Department, Hobart and Williams Smith College, 300 Pultney St., Geneva, NY 14456, USA; 3 Biomedical Sciences Department, Missouri State University, 901 S. National Ave., Springfield, MO 65897, USA

**Keywords:** programmed nuclear degradation, apoptosis, sirtuin, HDAC, Tetrahymena, ciliate, histone deacetylase

## Abstract

**Background:**

The NAD^+^-dependent histone deacetylases, known as "sirtuins", participate in a variety of processes critical for single- and multi-cellular life. Recent studies have elucidated the importance of sirtuin activity in development, aging, and disease; yet, underlying mechanistic pathways are not well understood. Specific sirtuins influence chromatin structure and gene expression, but differences in their pathways as they relate to distinct chromatin functions are just beginning to emerge. To further define the range of global chromatin changes dependent on sirtuins, unique biological features of the ciliated protozoan *Tetrahymena thermophila *can be exploited. This system offers clear spatial and temporal separation of multiple whole genome restructuring events critical for the life cycle.

**Results:**

Inhibition with nicotinamide revealed that sirtuin deacetylase activity in *Tetrahymena *cells promotes chromatin condensation during meiotic prophase, differentiation of heterochromatin from euchromatin during development, and chromatin condensation/degradation during programmed nuclear death. We identified a class I sirtuin, called Thd14, that resides in mitochondria and nucleoli during vegetative growth, and forms a large sub-nuclear aggregate in response to prolonged cell starvation that may be peripherally associated with nucleoli. During sexual conjugation and development Thd14 selectively concentrates in the parental nucleus prior to its apoptotic-like degradation.

**Conclusions:**

Sirtuin activity is important for several functionally distinct events requiring global chromatin condensation. Our findings suggest a novel role for sirtuins in promoting programmed pycnosis by acting on chromatin destined for degradation. The sirtuin Thd14, which displays physiological-dependent differential localization within the nucleus, is a candidate for a chromatin condensation enzyme that is coupled to nuclear degradation.

## Background

Class III histone deacetylases, known as sirtuins, are a large and ancient family of NAD^+^-dependent protein deacetylases that regulate a range of cellular processes. These phylogenetically conserved enzymes deacetylate both histone and non-histone targets. Originally based on the founding family member, yeast Sir2, molecular phylogenetic analyses have since revealed five sirtuin subclasses I-IV and U [1], which display diversity in subcellular localization and function [2,3]. For example, of the seven human sirtuin homologs, several reside in the nucleus where they have roles in genomic stability and cell proliferation. Others act in the cytoplasm on cytoskeletal targets or work in mitochondria to regulate energy metabolism and responses to oxidative stress [4,5]. Sirtuins in subclass I, which include human SIRT1-3 and yeast Hst2 and Sir2, commonly localize to the nucleus (with exception of Hst2) where they have various chromatin-related functions. For example, Sir2 regulates telomeric, cryptic mating-type, and rDNA silencing [6]. This diverse set of functions underlies numerous links between development, disease, and sirtuin activity reported in recent years [7,8].

Many sirtuin-linked cell abnormalities may relate to their roles in chromatin dynamics. To further probe these possibilities we turned to the single-celled protozoan *Tetrahymena thermophila*. This ciliate expresses eleven putative sirtuins, most are more closely related to sirtuins in humans than to those in yeasts [9]. *Tetrahymena *provides several advantages for chromatin dynamics studies. First, the cells harbor two nuclei with different chromatin characteristics. The "macronucleus" is transcriptionally active and contains primarily euchromatin, but undergoes widespread facultative heterochromatin formation during cell starvation [10,11]. Conversely, the "micronucleus" is transcriptionally silent and contains chromatin that is highly condensed into constitutive heterochromatin-like structures throughout vegetative growth [12]. This unique nuclear dimorphism facilitates study of factors that contribute to the differentiation and maintenance of euchromatin and heterochromatin states in the respective nuclei.

Second, nuclear differentiation into the dimorphic micronucleus and macronucleus during sexual conjugation involves multiple processes including DNA replication, DNA fragmentation and elimination, chromatin remodeling/differentiation, and nuclear degradation. These events are easily synchronized in a cell population and occur in a strict temporal order in only a subset of the post-zygotic nuclei (resulting from meiosis, fertilization, and mitosis). Half of the highly condensed, transcriptionally inert post-zygotic nuclei differentiate into transcriptionally active, euchromatic nuclei, while the other half remain inert. Another notable feature of *Tetrahymena *development is the programmed degradation of select nuclei at distinct points in the development pathway. Following meiosis, three of four gametes degrade in the posterior end of the cell. Later, the parental macronucleus degrades as newly differentiating macronuclei become transcriptionally active [12]. The latter degradation mechanism resembles that of caspase-independent apoptosis in higher organisms in several ways, including occurrence of chromatin condensation and production of oligonucleosome sized DNA fragments [13], but lacks other hallmarks such as the phosphorylation of H2A.X [14,15].

In this study we investigated the contribution of sirtuin deacetylase activity to chromatin differentiation and programmed nuclear degradation. We found that nicotinamide, a physiological sirtuin inhibitor [16,17], prevented normal progression of both of these processes. Furthermore, we identified one sirtuin (named "*Tetrahymena *histone deacetylase 14"; Thd14) that resides in nucleoli, in mitochondria, and in distinct nuclear sub-structures, all in response to different physiological conditions and stages of the life cycle. Intriguingly, Thd14 accumulated in chromatin-rich regions of the degrading macronucleus during the period of chromatin condensation that precedes global degradation.

## Results

### Nicotinamide treatment affects meiotic condensation, nuclear differentiation, and degradation of the macronucleus during development

**Figure 1:**
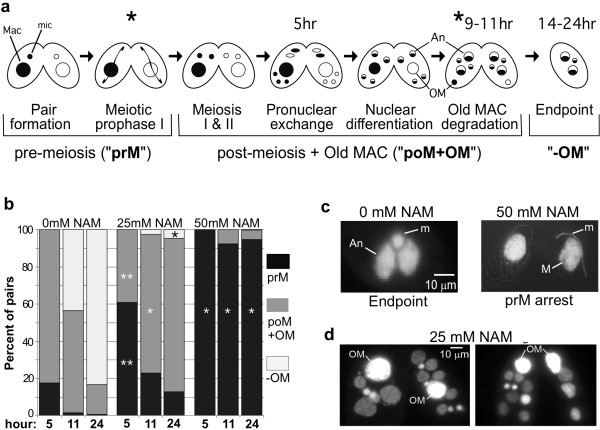
**Nicotinamide treatment causes arrest in meiotic prophase and prevents macronuclear degradation**. **a **Schematic of nuclear events in conjugation. Macronuclei are represented as large spheres, micronuclei as small spheres. Two mating types (white and shaded) were mixed to initiate conjugation. Asterisks represent the stages of arrest observed after nicotinamide treatment (coinciding with images in parts c and d). Hours represent times at which untreated conjugating cells (0 mM NAM) reach the given stage. Labels below the brackets indicate stages assessed in part b that correspond to different shades in the bar graph. "pOM+OM" are post-meiotic stages that still have a parental macronucleus (old macronucleus; OM); "-OM" is any stage after loss of the old macronucleus. An, anlagen (new macronuclei). **b **The average percentage of conjugating pairs at each of the three stages illustrated in part a by brackets: "PrM", cells in pre-meiosis (black); "pOM+OM", cells that contain an old macronucleus (dark gray); "-OM", cells that have lost the old macronucleus (light gray). Nicotinamide was added just after mixing (time zero of conjugation) and analyzed at 5, 11, and 24 hrs postmixing. At least 200 cells over two separate trials were counted for each time point and NAM concentration (each bar). ** P < 0.01 in a t-test when compared to 0 mM NAM at the same time point and stage of meiosis; *P < 0.05. **c **Representative images of DAPI-stained wild-type (0 mM NAM) and 50 mM NAM treated cells analyzed at 24-hrs into conjugation. M, macronucleus; An, anlagen; m, micronucleus. **d **DAPI images of 25 mM NAM-treated cells analyzed 24 hrs into conjugation. "OM", old (parental) macronucleus.

Phylogenetic analyses previously revealed eleven *Tetrahymena *proteins that possess sirtuin-type deacetylase domains [9]. This is a larger number than what most other organisms, including mammals, possess. Four of the proteins cluster in sirtuin subclass I, most members of which have nuclear functions. The potential contribution of sirtuin deacetylase activity to nuclear differentiation in *Tetrahymena *was examined by assessing nuclear development in conjugating cells treated with nicotinamide (NAM), a physiological sirtuin inhibitor [17,18]. Major chromatin changes during nuclear differentiation are accompanied by easily-distinguished changes in nuclear morphology. Cells of two different mating types were mixed (time 0) and immediately treated with 0, 10, 25 or 50 mM NAM. These concentrations were chosen based on those typically used for yeast and human cells (1-5 mM) and the fact that for *Tetrahymena*, other histone deacetylase inhibitors must be used at 5-10-fold higher concentrations for effects comparable to mammalian cells [19]. Nuclear morphology in NAM-treated conjugating pairs was compared to that of conjugating control cells (treated with buffer only), by DAPI staining and fluorescence microscopy at 2-3 hour intervals. Cells treated with 50 mM NAM arrested four hours into conjugation at meiotic prophase I (Figures 1a and 1b). At this stage micronuclei have drastically elongated and chromosomes have decondensed and assumed a "bouquet-like" arrangement [20] (Figure 1c, right panel). Based on the size and shape of prophase micronuclei in the NAM-arrested cells, the majority appeared to be in zygotene (stage III) and pachytene (stage IV) [21], even 24 hours later (Figure 1b). Cells treated with 10 mM (data not shown) and 25 mM NAM were able to progress through meiosis (Figure 1b) but displayed other downstream phenotypes described below. These phenotypes were detected in a greater fraction of cells treated with 25 mM NAM; thus, this concentration was used for further analyses.

Normally after fertilization, *Tetrahymena *zygotic nuclei undergo two mitoses. Two of the four resulting nuclei remain highly condensed, transcriptionally inert micronuclei, while the other two differentiate into new transcriptionally active macronuclei, called "anlagen" (Figure 1a). NAM-treated cells that were able to progress past meiosis contained 1-7 additional nuclei per conjugating pair after 24 hours, compared to untreated cells that had reached "endpoint" nuclear configuration by hour 16 (compare Figures 1c "Endpoint" and 1d). The additional nuclei in nicotinamide-treated cells likely resulted from one of two events: failure of three gamete pronuclei to degrade after meiosis, or additional mitoses of zygotic nuclei after fertilization. The former possibility was supported based on the following: 1) gamete pronuclei were rarely observed at the extreme posterior end of the cells where degradation occurs; 2) never were there fewer than four equally-sized nuclei observed in any cell of a pair during the early conjugation stages (normally three nuclei would appear much smaller when degrading at the posterior end; Figure 1a).

**Figure 2:**
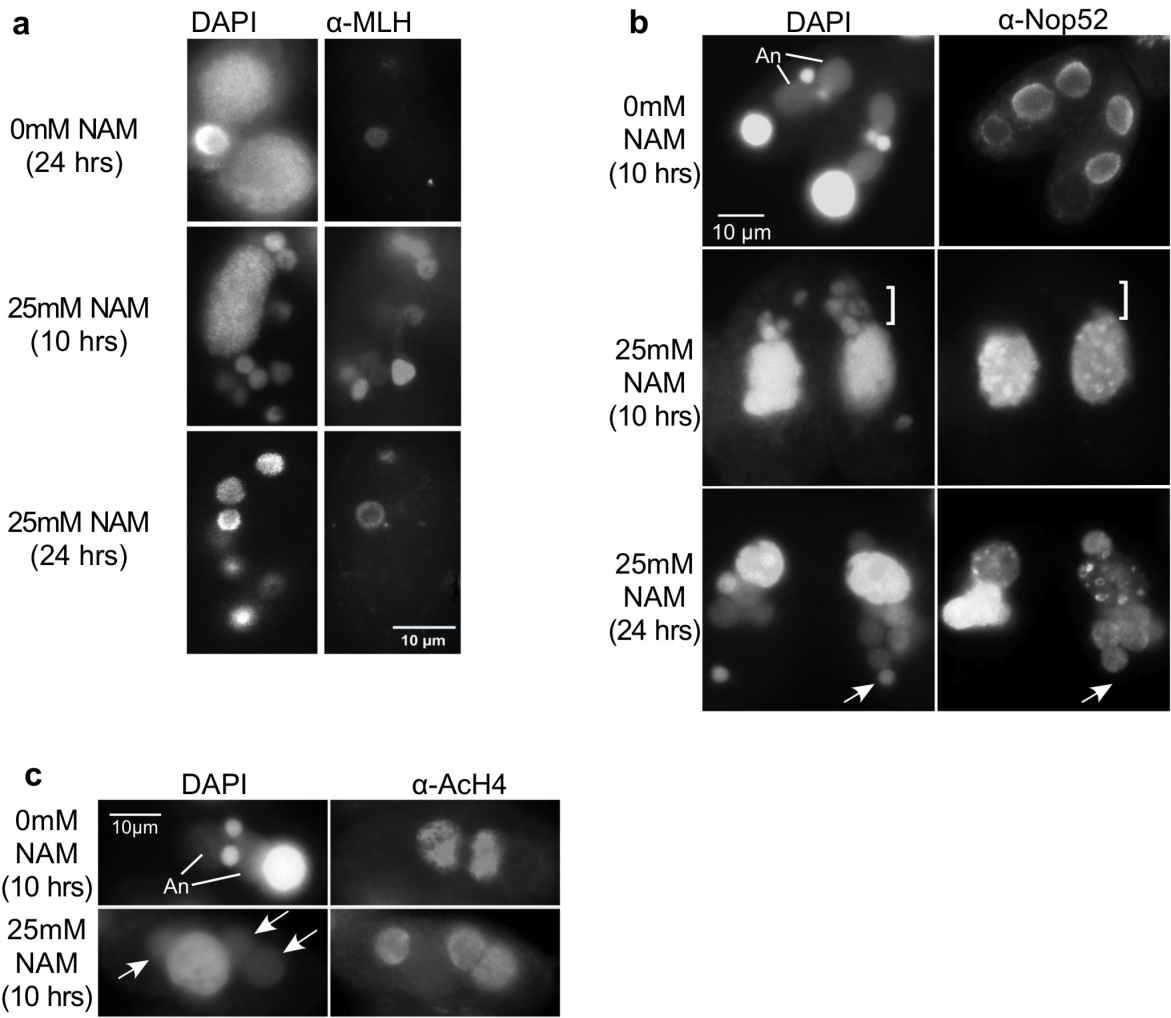
**Extra nuclei in NAM-treated cells acquire new macronucleus characteristics**. Cells were mixed in the absence (0 mM NAM) or presence (25 mM NAM) of nicotinamide and fixed with 2% paraformaldehyde at either 10 or 24 hrs into conjugation (post-mixing). Fixed cells were subjected to immunoblot analysis with **a **α-MLH (micronuclear linker histone; note that for the 24 hr NAM-treated sample a cell with an unusual α-MLH-positive micronucleus is shown for signal comparison), **b **α-Nop52 (brackets and arrows indicate examples of nuclear bodies that do not bind α-Nop52; "An", anlagen), or **c **α-acetylated H4 (α-AcH4) antisera (arrows indicate nuclear bodies that bind α-AcH4). All fixed cells were counterstained with DAPI.

Later in conjugation, the extra nuclei appeared to differentiate judging from nucleus size and intensity of DAPI staining (micronuclear DNA is entirely packaged into unacetylated heterochromatin and stains more intensely with DAPI, while anlagen chromatin decondenses, becomes highly acetylated, and stains less intensely with DAPI). The majority of nuclei in each cell were enlarged with diffuse chromatin, typical of anlagen morphology (Figure 1d). To further examine the differentiated status of chromatin, nuclei in NAM-treated conjugating cells were probed for the presence of the micronucleus-specific linker histone (MLH) by immunofluorescence with α-MLH antiserum. MLH, a hallmark of silent micronuclear heterochromatin, is selectively eliminated in nuclei destined to become new macronuclei when euchromatin differentiation and transcriptional activity is initiated [22]. Like all normal post-zygotic nuclei, NAM-treated nuclei contained MLH prior to differentiation (Figure 2a, 10 hrs). However, by 24 hrs after mixing, most cells (~90%) contained no MLH-positive nuclei; in approximately 10% of the cells one MLH-positive nucleus was observed (shown for signal comparison, Figure 2a, 24 hrs.). In contrast, untreated cells each contained one clear MLH-positive micronucleus (and two MLH-negative anlagen), reflecting normal development at 24 hrs after mixing. The loss of MLH in most nuclei of NAM-treated cells indicated that the extra nuclei were differentiating primarily into new macronuclei containing euchromatin (anlagen) at a ratio of 3-6 anlagen:1 micronucleus/cell (opposed to the normal 2 anlagen:1 micronucleus/cell). This interpretation was supported by the swollen appearance and weaker DAPI staining of these nuclei, two hallmarks of new macronuclei. Moreover, immunofluorescence studies showed that during their development, ~90% of the nuclei in NAM-treated cells acquired at least two other macronucleus-specific marks that appear in developing anlagen: the nucleolar protein Nop52 (Figure 2b), and acetylated histones (Figure 2c). Together, these results suggest that sirtuin activity is necessary for the maintenance of MLH and for the development of heterochromatin in half of the post-zygotic nuclei (those destined to differentiate into transcriptionally silent micronuclei). Furthermore, chromatin in additional nuclei (from sirtuin inhibition) appeared to differentiate toward transcriptional competency.

During the nuclear differentiation process, the old parental macronucleus migrates to the posterior end of the cell (Figure 1a) and degrades by a regulated mechanism that is coordinated with other developmental events. The mechanism shares features with apoptosis (chromatin condensation and oligonucleosome laddering) and autophagy (fusion of mitochondria and formation of an autophagosome) [13]. In our experiments, the old parental macronucleus failed to degrade in the majority of NAM-treated pairs. Cells retained their parental macronucleus even 24 hrs after mixing when new macronuclei had at least partially differentiated (Figure 1d), suggesting that sirtuin deacetylase activity was necessary for normal macronuclear degradation, which initiates prior to this stage. In addition, a larger fraction of cells retained their parental macronucleus if treated with NAM earlier in conjugation than shortly before its programmed degradation (see Additional File 1). Treatment immediately prior to the start of macronuclear degradation (~8 hrs) resulted in only ~33% cells retaining the old macronucleus (~2X more than untreated cells), compared to ~79% when treated at 2 hours post-mixing (~5X more than untreated cells).

**Figure 3:**
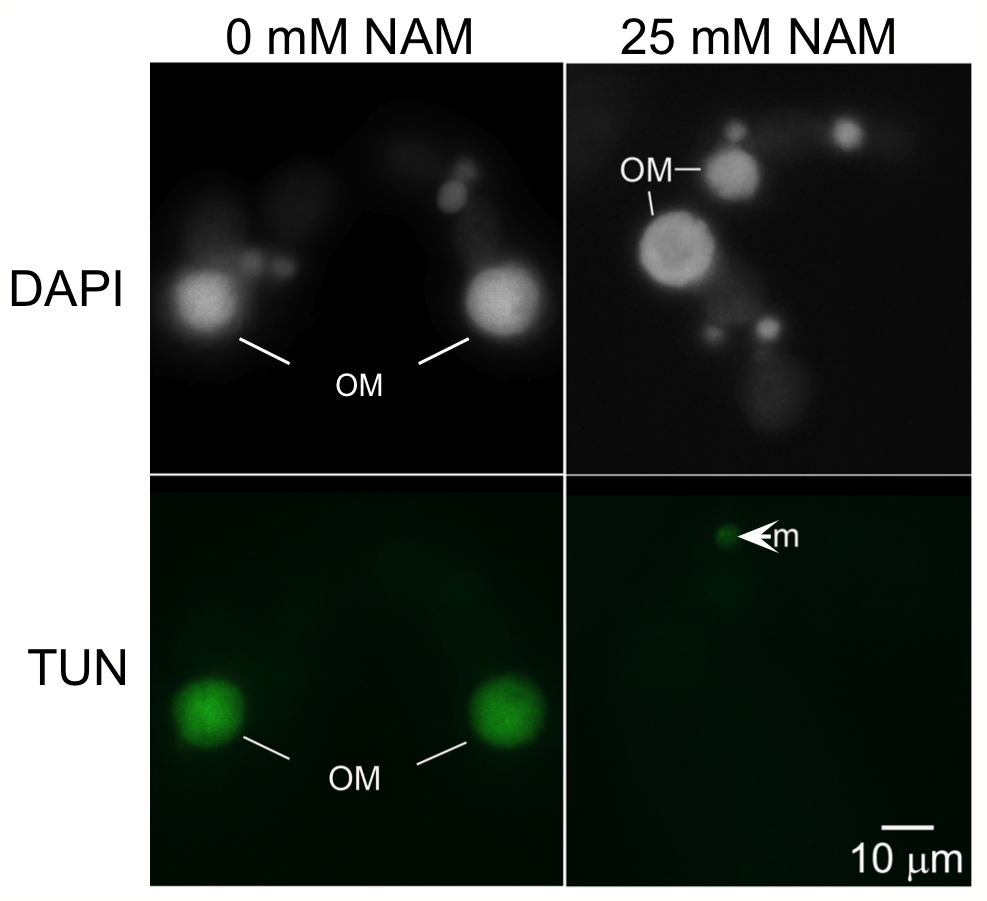
**Nicotinamide prevents degradation of the parental macronucleus during conjugation**. Just prior to mixing, cells were exposed to either 0 mM or 25 mM nicotinamide (NAM). At 10 hrs into conjugation (post-mixing), the cells were fixed in 2% paraformaldehyde, exposed to terminal deoxynucleotidyl transferase dUTP nick end labeling (TUNEL) reagent to assay for degraded DNA, and stained with DAPI prior to imaging. In cells exposed to NAM, the old macronuclei "OM" were TUNEL negative, while the OM of nontreated cells were TUNEL positive (green). "m", micronucleus. Note: A cell with an unusual TUNEL-positive micronucleus is shown for signal comparison in the NAM-treated sample.

To test whether NAM treatment compromised DNA degradation, conjugating pairs were subjected to a TUNEL (terminal deoxynucleotidyl transferase dUTP nick end labeling) assay to detect fragmented DNA. As expected, parental macronuclei in untreated conjugating cells were TUNEL positive 10 hrs into conjugation (Figure 3). In contrast, NAM-treated cells at the same stage with (obvious anlagen) contained a TUNEL-negative parental macronucleus. This result supported the idea that parental macronuclear DNA failed to degrade in sirtuin-inhibited cells. Occasionally, a single TUNEL positive micronucleus was observed later in conjugation, likely one that normally degrades at the end of conjugation (refer to Figure 1a). Here, this served as a positive signal comparison for "TUNEL-negative" nuclei (Figure 3, see arrow).

These results, which suggest a role for sirtuins in apoptotic-like nuclear degradation, prompted us to identify specific sirtuins that may contribute to the degradation process. The putative sirtuin named *Tetrahymena *Histone Deacetylase 14 (Thd14) was identified for further investigation due to its localization to the parental macronucleus in preliminary studies.

### Thd14 resembles a class 1b sirtuin

**Figure 4:**
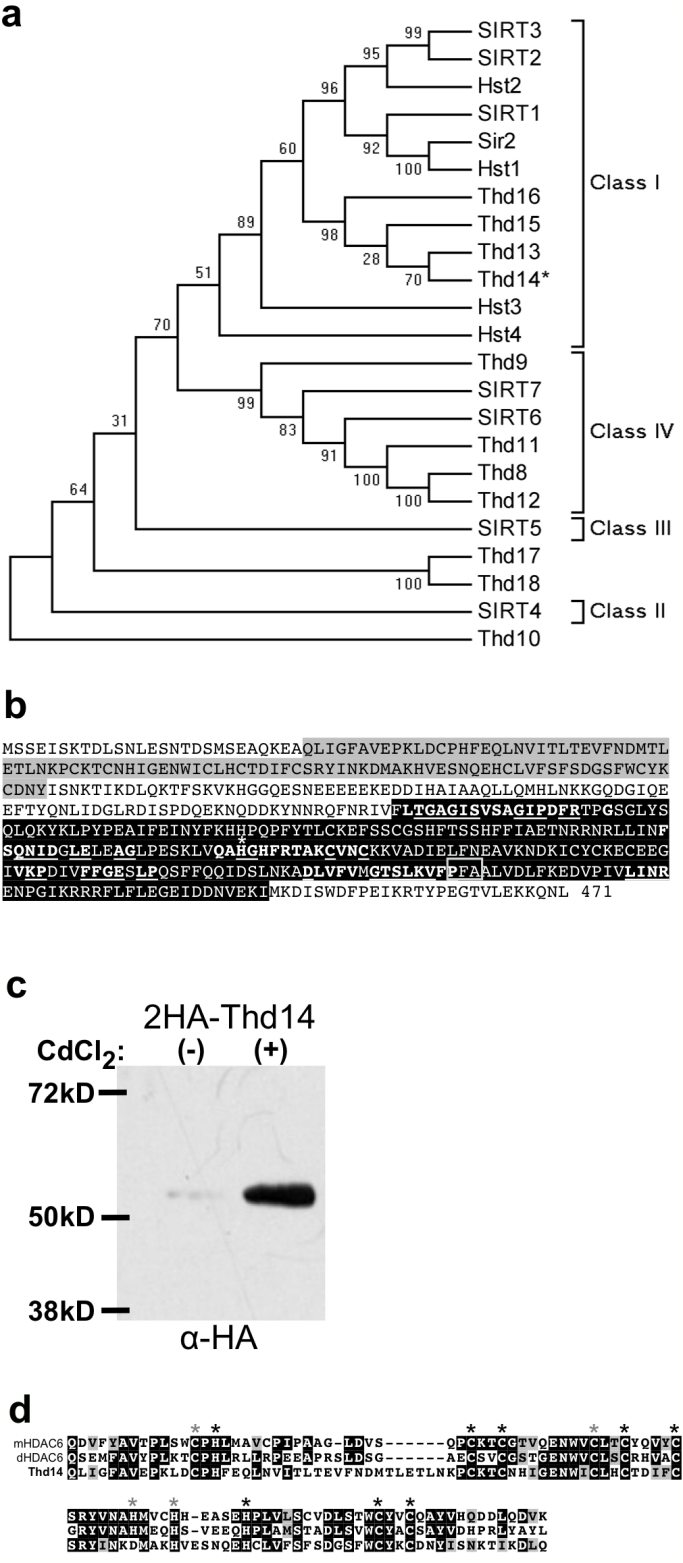
**Thd14 resembles a class 1b sirtuin**. **a **Phylogenetic tree comparing the primary structure of the *T. thermophila *sirtuins including Thd14 (asterisk) with that of the yeast sirtuins (Sir2 and Hst1-4) and the seven human sirtuins (SIRT1-7) (unweighted-pair group methods using average linkages). The scale (x-axis) represents evolutionary distance as calculated using the Poisson correction method, with the units of number of amino acid substitutions per site. **b **Amino acid sequence of Thd14 translated from the gene sequence. The predicted zinc finger domain is highlighted in grey and the sirtuin domain is highlighted in black. The motifs of conserved amino acids present within the sirtuin core domain are in boldface type. Underlined letters indicate intraclass-conserved residues, used for sirtuin identification. The asterisk denotes the critical catalytic histidine residue in the 'HG' motif that is strictly conserved in all known sirtuins. Boxed is a sub-motif specific to class 1b sirtuins. **c **Cells expressing HA-Thd14 were grown in the absence "(-)"or presence "(+)" of 1 μg/mL CdCl_2 _for 2 hrs to induce expression from the *MTT1 *promoter. Total cellular proteins were resolved by SDS-PAGE, transferred to nitrocellulose membrane, and subjected to immunoblot analysis using α-HA antiserum. **d **Amino acid alignment of the UBP-type zinc-finger domain from Thd14 with similar domains from murine and *Drosophila *HDAC6 (mHDAC6 and dHDAC, respectively). Asterisks mark conserved Cys and His residues; black asterisks denote those in HDAC6 that are essential for ubiquitin binding [24]. Conserved residues are shaded in black (for identical residues) and grey (for chemically similar residues).

Amino acid sequence comparisons of open reading frames predicted by the *Tetrahymena *Genome Database (TGD; http://www.ciliate.org) identified gene (TTHERM_00526990), hereafter called *THD14*, as one of eleven putative sirtuins encoded in the genome [9]. A phylogenetic tree constructed with all human, yeast, and putative *Tetrahymena *sirtuins showed that Thd14 has strongest similarity to the class 1 sirtuins, along with three other *Tetrahymena *proteins (Figure 4a). Amino acid alignments between the sirtuin core domain of Thd14 and those of human class I sirtuins (SIRT1, SIRT2, and SIRT3) show strong homology (SIRT1: 45% identical, 62% similar; SIRT2: 44% identical, 61% similar; SIRT3: 40% identical, 60% similar; see Additional File 2 for a multiple sequence alignment).

Multiple Expressed Sequence Tags (ESTs) spanned the majority of the computationally-predicted coding sequence for Thd14 (*Tetrahymena *Genome Database; http://www.ciliate.org). The expected coding sequence was confirmed using reverse transcriptase-PCR to amplify cDNA with primers spanning the predicted START and STOP codons, followed by cloning and sequencing of the products. Resulting THD14 cDNA sequence was consistent with the computationally predicted coding sequence. The gene contains one intron and encodes a 471 amino acid protein with two domains: a sirtuin deacetylase domain and a UBP-like zinc finger domain (Figure 4b). The predicted protein molecular mass of 54.1 kD was experimentally confirmed by immunoblot analysis of lysates from cells expressing 2 × HA epitope-tagged Thd14. Anti-HA antiserum detected a protein of ~57 kD (54 kD plus ~3 kD 2 × HA tag) according to its mobility in SDS-PAGE (Figure 4c).

Computational analysis of Thd14 amino acid sequence revealed a sirtuin "core domain" (Figure 4b, black shading) with typical sirtuin motifs (Figure 4b, bold white lettering) and highly conserved (underlined) catalytic domain residues [1,9]. Importantly, Thd14 contains the critical catalytic histidine residue in the 'HG' motif that is strictly conserved in all known sirtuins (asterisk, Figure 4b). Immunoprecipitated HA-Thd14 catalyzed the deacetylation of acetylated N-terminal histone peptide substrates, thus confirming it as a functional histone deacetylase (Additional File 3). The presence of specific intraclass-conserved residues further supported assignment of Thd14 as a class I sirtuin. In particular, the PFA sub-motif is specific to class Ib enzymes, as is the alanine preceding the HG motif (Figure 4b, boxed) [1].

One unique feature of Thd14 is an amino terminal zinc-finger domain of the ubiquitin protease (UBP)-type superfamily, commonly found on ubiquitin hydrolase enzymes (Figure 4b, grey shading). Interestingly, the highest domain homology is with a UBP-type domain found on the murine class II histone deacetylase HDAC6 (36% identical, 55% similar; Figure 4d). Named a Polyubiquitin Associated Zinc finger (PAZ) domain, it coordinates 3 Zn^2+ ^ions to bind with high affinity to polyubiquitin protein modifications [23-25]. This domain on Thd14 contains 11 out of the 12 highly conserved cysteine and histidine residues used to coordinate the Zn^2+ ^ions (Figure 4d, all asterisks). Of those residues, the domain on Thd14 contains all that are absolutely required for ubiquitin binding (2 histidines and 6 cysteines) [24] (Figure 4d, indicated by black asterisks). Thd14 is the only one of the eleven *Tetrahymena *sirtuins that contains a PAZ domain.

### *THD14 *transcription peaks in early conjugation

**Figure 5:**
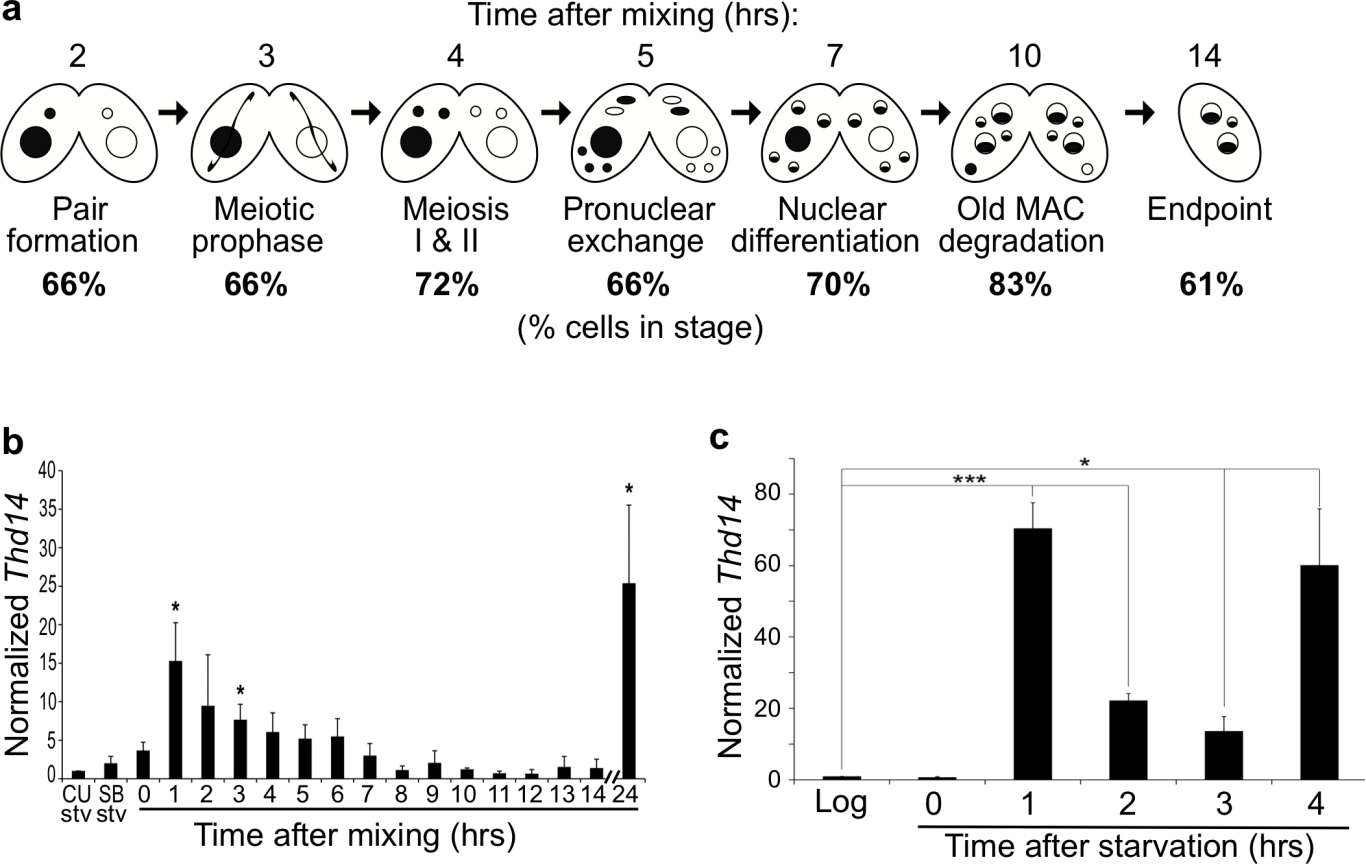
**Thd14 expression peaks in pre-meiotic stages**. **a **Synchrony of conjugating cells used for cDNA analyses. Cells of two different mating types were mixed to initiate conjugation, and samples of the conjugating culture were dried and stained with DAPI for visualization of stages by fluorescence microscopy every hour. Hours post mixing are indicated ("hrs") above the corresponding stages illustrated. The percentage of conjugating pairs in each stage at the corresponding time is indicated below the diagram. The last stage is percentage of single cells. **b **Relative *THD14 *expression of starved CU428 (CU Stv) and SB1969 (SB Stv) as well as during conjugation (0-14 and 24 hours after mixing) was determined using quantitative real-time PCR. *THD14 *expression was normalized to *CYP1 *expression (*THD14/CYP1*) and the levels of *THD14 *expression were set relative to the starved CU428 (normalized values of each sample/CU428). Error bars represent the standard deviation of three separate trials and asterisks indicate time points that had a significant (p < 0.05) increase in expression of *THD14 *compared to either the starved CU428 cells (CU Stv) or immediately after mixing (0 hrs). *THD14 *mRNA levels increased early in conjugation at (1-3 hours) and late (24 hours) after completion of conjugation. **c **Relative *THD14 *expression of logarithmically growing CU428 ("Log") as well as during starvation (hours 0 through 4) was determined using quantitative real-time PCR. *THD14 *expression was normalized to that of *BTU1 *and the levels of *THD14 *expression were set relative to logarithmically growing cells. Error bars represent the standard deviation of three separate trials and asterisks indicate time points that had a significant (* p < 0.05, *** p < 0.005) increase in expression of *THD14 *compared to logarithmically growing cells.

*THD14 *expression throughout the *Tetrahymena *life cycle was examined. Two different mating types were starved (CU428 and SB1969), mixed to initiate conjugation, and allowed to conjugate over a 24-hour period. Population synchrony was monitored at regular intervals by determining the fraction of cells in each developmental stage (Figure 5a). Throughout the entire conjugation time course, 60 to 70% of the cells were tightly synchronized and an additional 20 to 25% of the culture was within 60 minutes of this primary synchronized population. Reverse transcriptase-PCR (data not shown) and qRT-PCR using cDNA made from the conjugating cells at 1-hour intervals (and from mid-logarithmic growing cells) was performed. *THD14 *transcription was moderate in growing and 24-hour starved cells and elevated in early (pre-meiotic) stages of conjugation. Then, Thd14 expression slowly decreased over subsequent intervals, except at 24 hrs after mixing (once cells have all achieved "endpoint"), where the highest expression level was observed (Figure 5b). These results were consistent with those from microarray experiments, which also show that the 24 hour peak initiates at 18 hrs http://tged.ihb.ac.cn[26]. Together, these results suggest that *THD14 *transcription is regulated according to physiological state and developmental stage, and raises the possibility of a role for this sirtuin in pre-meiotic events.

In other cell types, select sirtuin activities are induced by starvation. To examine Thd14 expression during starvation, mRNA was quantified by qRT-PCR over a 4 hour period following nutrient depletion. Relative to vegetatively growing cells, a dramatic increase in expression was observed immediately with starvation initiation, and levels remained high over the interval tested (Figure 5c). This result raised the possibility that Thd14 has a starvation-specific function that is rapidly induced with nutrient depletion.

### Thd14 is in mitochondria and nucleoli during vegetative growth, and aggregates during starvation

**Figure 6:**
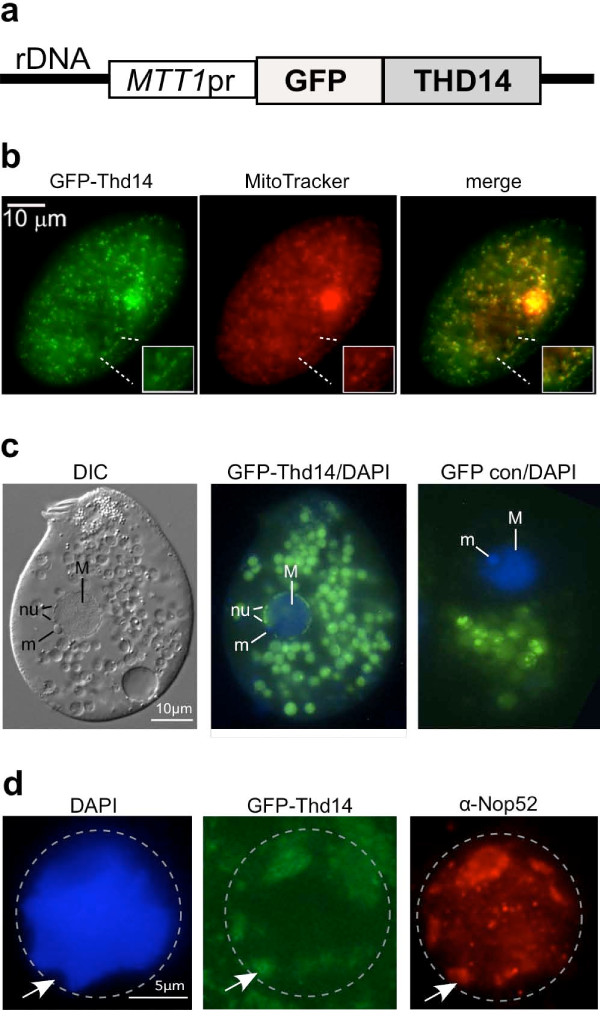
**Thd14 resides in mitochondria and nucleoli during growth**. **a **Line drawing schematic of the construct engineered on an rDNA vector for expression of GFP-Thd14 fusion protein from the metallothioneine promoter (*MTT1*pr). During conjugation, the vector is processed and amplified as a small linear rDNA chromosome. **b **Fluorescence microscopy image of a live cell showing GFP-Thd14 localizing to mitochondria (stained with Mitotracker) near the surface of the cell. Inlays show magnification of one mitochondrial-dense region. The larger region of intense staining is rarely observed in other cells and thus considered an artifact. **c **Differential interference contrast (DIC) imaging of a live cell in vegetative growth shows locations of nuclear structures, including the micronucleus (m) and multiple nucleoli (nu) positioned around the macronuclear (M) periphery. Fluorescence microscopy shows a merge of DAPI imaging with GFP-Thd14 in nucleoli (center panel). "GFP con/DAPI" is the GFP only control (no fusion) with a DAPI image overlay. Green spheres outside the nucleus are probably food vacuoles, a common artifact observed with high GFP-protein expression. Live cells were concentrated and mounted in 2% methylcellulose for observation. **d **Fluorescence microscopy on a paraformaldehyde-fixed cell nucleus (bounded by dashed circle) showing co-localization of GFP-Thd14 and Nop52 by immunofluorescence in regions staining poorly with DAPI (arrow indicates an example).

To examine localization of Thd14, Green Fluorescent Protein (GFP) was fused to the amino terminus. The fusion gene, GFP-THD14, was carried on a high-copy *Tetrahymena *rDNA vector [27] and expressed from the inducible metallothionein 1 (*MTT1*) promoter (Figure 6a) [27,28]. In mid-logarithmic growing cells, GFP-Thd14 was detected in mitochondria, which were marked with fluorescent Mitrotracker dye (Figure 6b). GFP-Thd14 was also detected in the multiple nucleoli positioned around the macronuclear periphery (~90 nucleoli per nucleus; Figure 6c), which were illuminated by differential interference microscopy (labeled "nu" on DIC image). Food vacuoles in the cytoplasm, a common fluorescent artifact from over-expression of GFP tagged proteins (shown by "GFP con/DAPI" panel), were ignored in this analysis. Consistent with nucleolar localization, immunofluorescence revealed that the nucleolar protein Nop52 [29] co-localized to regions containing GFP-Thd14 (Figure 6d). Furthermore, these regions with GFP-Thd14 stained only weakly with DAPI, a common characteristic of nucleoli.

**Figure 7:**
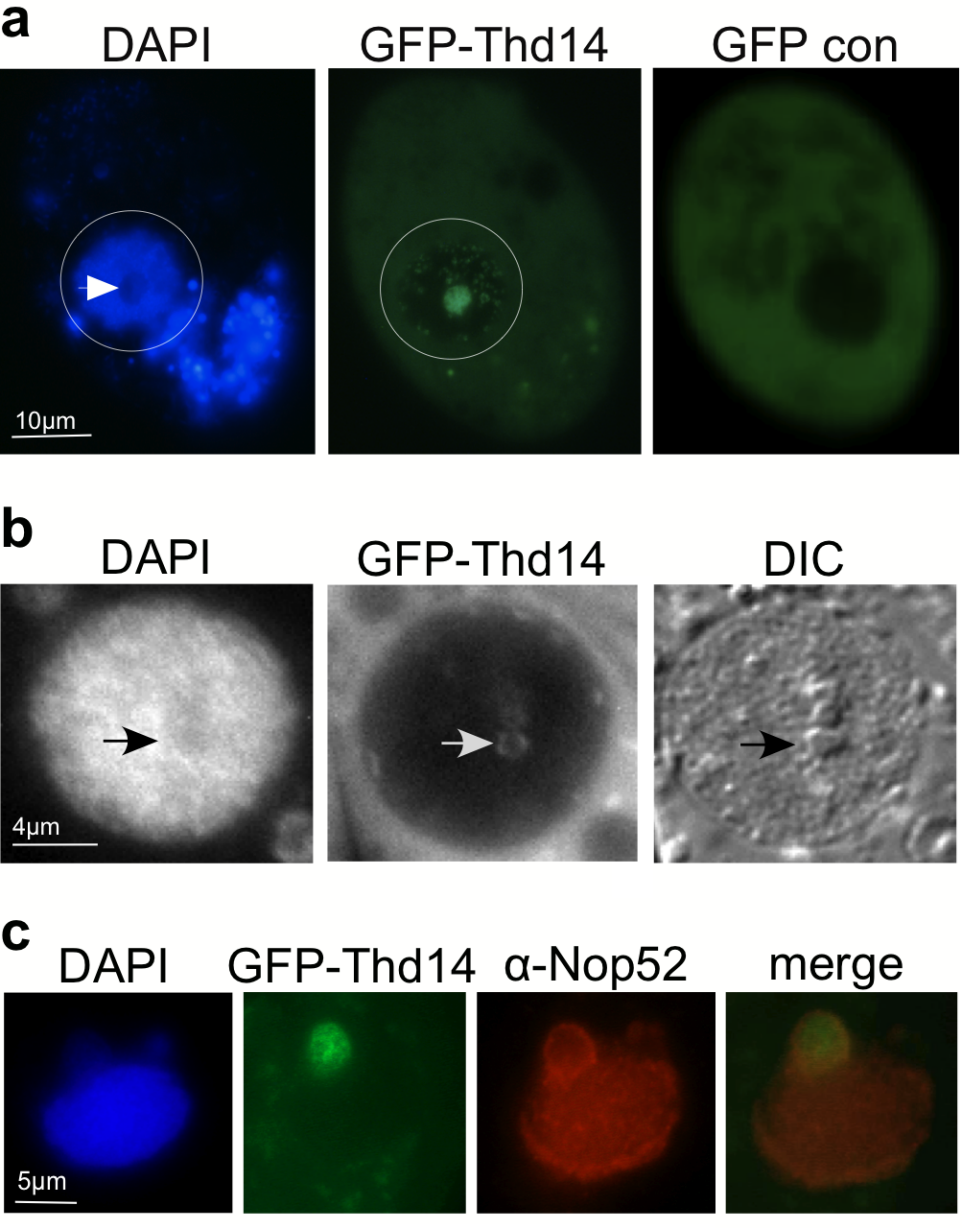
**Thd14 resides in a large aggregate during starvation**. **a **A starved live cell expressing GFP-Thd14 visualized by fluorescence microscopy. The macronucleus is within the circled regions. Staining outside the nucleus is in food vacuoles. White arrow points to a DAPI poor region where GFP-Thd14 aggregates. "GFP con" is the negative control expressing only GFP (no fusion with Thd14). **b **DAPI, GFP and DIC images of a macronucleus in a live cell. Masses inside the macronucleus observed by DIC microscopy (arrow) are ringed by GFP-Thd14 and stain weakly with DAPI, both observed by fluorescence imaging. **c **A macronucleus showing Nop52 ringing around the large aggregate of GFP-Thd14 by immunofluorescence.

We next tested whether GFP-Thd14 remained in nucleoli under conditions that alter rDNA metabolism. With prolonged nutrient starvation, *Tetrahymena *nucleoli aggregate to form larger masses around the nuclear periphery [30] coincident with a global decrease in transcription of rDNA and many protein-coding genes. After 18-20 hours of starvation, GFP-Thd14 was still present in the nucleoli in the majority of cells. However, the most intense fluorescence concentrated in a single large aggregate within the macronucleus (Figure 7a) that was visible by 30 minutes after nutrient depletion (quickly following the increase in *THD14 *expression; Figure 5c), and reached maximum size 4 hours later. Of the cells showing nucleolar fluorescence around the periphery (~85%), approximately half (45/96) contained the larger aggregate. The aggregate of fluorescence appeared as a clustering of multiple small rings, suggesting sub-structural organization of Thd14 within the larger aggregate (Figure 7b). As well, DAPI staining was weaker or absent from the concentration of GFP-Thd14. DIC imaging mapped Thd14 aggregates to distinct structures within the nucleus (Figure 7b, right panel). Immunofluorescence with anti-Nop52 antiserum revealed that Nop52 ringed the entire Thd14 aggregate (Figure 7c) indicating a relationship to nucleoli.

### Thd14 concentrates inside the macronucleus immediately prior to programmed degradation

**Figure 8:**
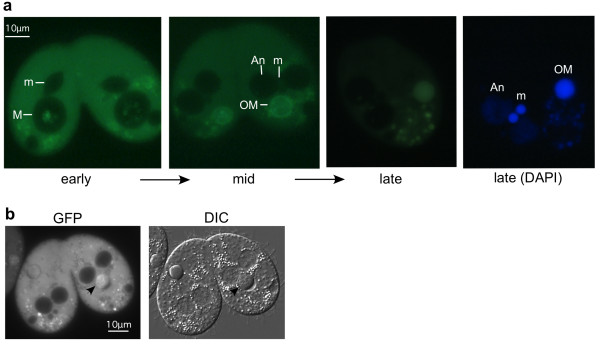
**Thd14 concentrates inside the macronucleus immediately prior to pycnosis**. **a **Fluorescence microscopy images of live conjugating pairs showing localization of GFP-Thd14 at three points in conjugation designated "early" (before meiosis), "mid" (after meiosis, mid-development of anlagen) and "late" (after pair separation, prior to macronuclear reabsorption). M, macronucleus; An, anlagen; m, micronucleus. Live cells were concentrated and mounted in 2% methylcellulose. **b **DIC and fluorescence microscopy images of a live conjugating pair showing GFP concentration over condensed chromatin regions in degrading macronuclei (arrows). Cells were prepared as in part a.

To assess Thd14 distribution during development and nuclear differentiation, cells expressing GFP-Thd14 were starved and mixed with wild type cells of a different mating type to initiate sexual conjugation. Samples of conjugating pairs were examined by fluorescence microscopy at regular intervals throughout the course of conjugation (16 hours) and 24 hours after initiation (Figure 8a). In early conjugation (2-5 hrs, micronuclear meiosis and mitosis) the rings in the GFP-Thd14 focus became more pronounced, then diffused throughout the macronucleus. By mid-conjugation (5-8 hrs) at stages immediately preceding old macronucleus pycnosis and degradation, GFP-Thd14 became increasingly and selectively concentrated within the old macronucleus, but remained absent from the young, developing new macronuclei (Figure 8a). Within the degrading nucleus, Thd14 localized to the area occupied by pycnotic heterochromatin within the boundary of the nuclear envelope as revealed by DIC microscopy (Figure 8b).

## Discussion

Sirtuins are known to influence chromatin dynamics related to gene silencing, DNA repair, and maintenance of chromosome structural features such as telomeres. In this study we examined processes involving chromatin dynamics that are easily synchronized and monitored using *Tetrahymena *conjugation. Sirtuin inhibition with nicotinamide (NAM) affected three events requiring significant changes to global chromatin structure: (1) meiotic chromosome condensation, (2) differentiation of transcriptionally inert nuclei, and (3) programmed nuclear degradation.

During meiotic prophase, the germline micronucleus elongates ~50-fold resulting in a string-like "crescent" nucleus. Chromosomes decondense and assume a bouquet-like formation for homologous pairing and recombination, then re-condense prior to metaphase I [31,32]. With 50 mM NAM treatment, decondensation and elongation was unaffected (normal numbers of fully elongated crescents were observed), but re-condensation appeared incomplete; the majority of cells arrested with partially elongated micronuclei (Figure 1b). The class I sirtuin, SIRT2, was previously implicated in promoting chromosome condensation during mitosis in human cells by deacetylating histone H4 [33]. However, evidence for sirtuin roles in meiotic chromatin dynamics was lacking prior to this study.

Cells in a lower NAM concentration proceeded through meiosis, but displayed other chromatin differentiation defects. First, more than four post-zygotic nuclei were observed in most cells. Of these nuclei, all (or most in some cells) initiated new macronucleus chromatin development involving loss of micronuclear linker histone (MLH) (Figure 2a), and gain of both Nop52 (Figure 2b) and histone acetylation (Figure 2c). The ratio of differentiating macronuclei to micronuclei was 3-6:1 instead of the normal 2:1 in most pairs (Figures 1d and 2-all parts). These data suggest that sirtuins normally promote the retention of MLH and development of heterochromatin in micronucleus-destined nuclei. Our observations suggest that the abnormally large number of nuclei in sirtuin-inhibited cells resulted from failure of gamete degradation prior to fertilization (3 of the 4 gametes normally degrade), an aberrant event that could be mechanistically related to the failure of parental macronuclear degradation observed later in conjugation. This idea is consistent with a previous observation that halting macronuclear degradation with PI-3 kinase inhibitors led to the retention and reprogramming of pronuclei to differentiate into micro- and macronuclei [34]. Our results indicate that the retained extra nuclei are capable of differentiating from transcriptionally silent to transcriptionally active nuclei, and that this pathway is chosen (over maintenance of the silent state) in the absence of sirtuin activity.

Unlike normal cells that lose their parental macronucleus by 16 hours into conjugation, NAM-treated cells retained a macronucleus that exhibited little to no DNA degradation (Figure 3). Programmed macronuclear degradation is thought to proceed through an apoptosis-like, caspase-independent mechanism that is dependent on endonuclease G activity and apoptosis inducing factor (AIF) stored in mitochondria [35,36]. There is a well-described relationship in which chromatin condensation precedes DNA fragmentation in apoptosis [37]; one that also applies to programmed nuclear death in *Tetrahymena *[35,38,39], but the mechanistic details underlying these chromatin changes in any system are poorly understood. In *Tetrahymena*, macronuclear DNA first condenses to less than half its original volume by 14 hours of conjugation, followed by global degradation of chromatin and resorption by the autophagosome [13,35,39]. This degree of condensation was inhibited in the majority of NAM-treated cells (Figure 1b). Given that DNA degradation also failed in these nuclei (Figure 3), sirtuin activity may be necessary for the chromatin condensation step of nuclear degradation, a prerequisite to global degradation. Interestingly, sirtuin inhibition did not affect the global deacetylation of histone H4 in parental macronuclear chromatin during pycnosis (Figure 2c), but it may be targeting other histones or nonhistone targets to mediate condensation. Our NAM treatment time course (Additional File 1) suggests sirtuin involvement in an earlier degradation step, possibly involving initial signaling. Normally, commitment of nuclei to new macronuclear development at 6-7 hours into conjugation triggers destruction of the old parental macronucleus, which involves its migration to the posterior region of the cell [38,40]. In our study, although NAM-treated nuclei committed to anlagen development, parental macronuclei failed to migrate in ~30% of cells (Figures 1d and 3) consistent with possible disruption of the degradation "triggering" event.

Sirtuins are involved in signaling pathways preventing apoptosis and cellular senescence in other organisms [41], but no direct action on chromatin destined for degradation has been described. Although it is possible that the NAM treatment in our study inhibited nuclear degradation through blocking sirtuin-mediated signaling pathways, we present evidence that at least one *Tetrahymena *sirtuin, Thd14, could act directly on chromatin destined for degradation. Thd14 selectively accumulated in the parental macronucleus (not in anlagen) at the initial stage of chromatin condensation (~8 hrs), and in later stages mapped precisely over the region of condensed chromatin within the macronuclear envelope (Figures 8a and 8b). The mechanism for Thd14 accumulation will be the focus of future studies. We speculate that Thd14 plays a role in macronuclear degradation by directly modifying histones. While our HDAC assay confirmed that Thd14 is capable of deacetylating histones *in vitro *(Additional File 3), it is also possible that this enzyme may target other substrates under biologically relevant conditions. Other than histone H2AX and H2B phosphorylation, little is known about other modifications to apoptotic chromatin, but histone deacetylation appears critical in at least some cases. Deacetylation of yeast histone H2B by the Hos3 HDAC (class II enzyme) is required for apoptosis [42], and apoptotic condensation in leukemia cells was linked with global histone deacetylation [43], but the deacetylases remain unknown. Given their involvement in heterochromatin formation at various genomic loci [6], sirtuins are reasonable candidates for apoptotic chromatin modifiers. Our combined results from NAM-inhibition and Thd14 localization provide the first evidence that a sirtuin(s) acts on chromatin destined for degradation.

One intriguing feature of Thd14 is its zinc finger domain, which is unusual to find on a sirtuin enzyme. Its strong homology to the PAZ domain on HDAC6 known to bind ubiquitin [23-25], suggests that Thd14 may interact with ubiquitin or ubiquitinated proteins, especially since it contains all of the essential binding residues [24] (Figure 4d). Since ubiquitin plays a major role in apoptosis and labels proteins for degradation, the PAZ and sirtuin domains of Thd14 may collaborate to make essential apoptotic modifications. Although a single protein with both a sirtuin and PAZ domain has not been identified in higher organisms, Thd14 may combine functions that higher-order organisms have evolved to handle with separate, more specialized proteins. To assess these possibilities, function of the putative PAZ domain and its potential role in nuclear degradation will be the subject of future studies.

Results in this study showed that Thd14 targeting was dependent on physiological state. In the nucleus of vegetatively growing cells, Thd14 resided primarily in the multiple nucleoli positioned around the nuclear periphery, and in mitochondria. Sirtuins in other organisms are known to act at nucleolar loci where they stabilize rDNA repeats and factor into RNA polymerase I transcription and rDNA silencing [44-47]. Interestingly, nutrient starvation dramatically increased *THD14 *expression (Figure 5c), and caused Thd14 protein to concentrate into a prominent focus, or aggregate, inside the nucleus (Figure 7a). Like nucleoli, this aggregate was often associated with the nuclear periphery and stained weakly with DAPI (Figure 7a, left panel). However, it was much larger than the typical nucleolar aggregates previously observed by electron microscopy [48,49], and was ringed only around the periphery by the nucleolar protein Nop52 (Figure 7c). Previous work showed that under certain gene over-expression conditions (PML and p53) the human Sir2 homolog, SIRT1, is recruited to discrete nuclear foci with promyelocytic leukemia (PML) protein, in "PML bodies" where it deacetylates proteins such as p53 [50]. Similarly, Thd14 may be sequestering with other nuclear proteins, possibly those involved in regulating cellular response to starvation stress.

Our study raises the intriguing possibility that the sirtuin Thd14 is specialized for the formation of irreversible heterochromatin functionally linked to parental macronucleus degradation, but not for reversible, facultative heterochromatin like meiotic chromatin in micronuclei. Despite the elevated expression in pre-meiotic stages, Thd14 did not localize to micronuclei at any point before or during meiosis, and formation of these two types of heterochromatin appears mechanistically different with respect to to histone modifications [51]. Especially intriguing is that localization results suggest additional roles for Thd14 in nucleoli and mitochondria. Whether they relate to nuclear degradation mechanisms will be a focus of future studies. In one possible model consistent with the macronuclear autophagy process, Thd14 is delivered specifically to the parental macronucleus from mitochondria that fuse with the nucleus prior to degradation, a mechanism previously shown to deliver other degradation factors such as endonucleases and AIF [35,52]. Such a model would explain the increased concentration of parental macronuclear Thd14 in the absence of increased expression (Figure 5b). Regardless of its concentration mechanism, we expect future work to define a role for Thd14 in promoting or coordinating macronuclear autophagy.

## Conclusions

The sirtuin inhibitor nicotinamide prevents meiotic chromosome condensation, and normal progression of both chromatin differentiation and programmed nuclear degradation during development in *Tetrahymena*, all of which depend on global chromatin condensation. One sirtuin, Thd14, resides in mitochondria, nucleoli, and in distinct nuclear sub-structures depending on physiological conditions and stages of the cell's life cycle. Notably, Thd14 accumulates in chromatin-rich regions of the degrading macronucleus during the chromatin condensation stage preceding global degradation. Together, the NAM-inhibition and Thd14 localization studies suggest the first evidence that sirtuins act on chromatin destined for degradation in apoptotic nuclei.

## Methods

### Bioinformatics

The amino acid sequences of the eleven *Tetrahymena *sirtuins (Thd8 through 18), yeast Sir2 and Hst1 through 4, and human SIRT1 through 7 were aligned using Tree-based Consistency Objective Function For alignment Evaluation (TCOFFEE; http://www.ebi.ac.uk/t-coffee/). The molecular phylogeny was then evaluated using Multiple Sequence Alignment-CLUSTALW [Kyoto University Bioinformatics Center http://www.genome.jp/tools/clustalw/]. The CLUSTAL protein alignment was performed using a gap open penalty of 10, a gap extension penalty of 0.05, a hydrophobic gap, no weight transition, and a BLOSUM weight matrix. Distances were computed using the Poisson Correction Distance method in Molecular Evolutionary Genetic Analysis (MEGA) software version 4.0 (MEGA4) [53]. The unweighted-pair group method using an average linkages tree was constructed from the matrix of distances according to the model using MEGA4, and the robustness of the tree topology was tested with 1,000 bootstrap replicates. Sirtuin core domains were identified using ExPASY-PROSITE [Swiss Institute of Bioinformatics http://prosite.expasy.org/].

### Statistical analyses

For qRT-PCR: results are indicated as the mean ± standard deviation. Statistical significance in qRT-PCR and NAM treatment studies was determined by independent, two tailed *t*-tests in Microsoft Excel to compare differences between two groups. p-values of < 0.05 were considered significant.

### Strains and cell culture conditions

*Tetrahymena thermophila *strains CU727 (btu1-1::btu1-1M350K/btu1-1::btu1-1M350K; ory-r, tax-s, V), CU724 (btu1-1::btu1-1M350K/btu1-1::btu1-1M350K, *chx1-1/chx1-1*; cy-r, mp-r, ory-r, tax-s, VII), CU427 (*chx1-1/chx1-1 CHX1*; cy-s, VI), and CU428 (*mpr1-1/mpr1-1 MPR1*; mp-s, VII) provided by the National *Tetrahymena *Stock Center at Cornell University, were used as wild-type strains. For all experiments, *Tetrahymena thermophila *strains including the strain expressing GFP-tagged Thd14 (GFP-Thd14) were grown in 2% PPYS medium (0.02 g/mL proteose peptone, 0.002 g/mL yeast extract, and 0.03 mg/mL sequestrine) containing 2X PSF (penicillin, streptomycin, and fungizone; Gibco-BRL) with shaking (150 rpm) at 30°C, until mid-logarithmic phase (1x10^5 ^to 3x10^5 ^cells/ml). The cells were starved in 10 mM Tris-HCl (pH 7.4) for 14 to 24 hrs at a density of 3x10^5 ^cells/ml at 30°C without shaking.

For nicotinamide (NAM) treatment experiments, cells of two different mating types (CU428 and CU427) were mixed together after starving each strain for 18-24 hrs in 10 mM Tris (pH 7.5). Cells were treated with 50 mM NAM at the time of mixing ("0 hrs"), and with 25 mM NAM at 0, 2, 4, or 6 hrs after mixing. For analysis of developmental morphology, at least 200 cells were counted for each time point and NAM concentration.

### HDAC activity assay

Whole cell protein lysates were generated from 7 × 10^6 ^cells of both CU427 (wild type) and cells expressing HA-Thd14 by vortexing for 1 min with acid-washed glass beads (Sigma) in 350 μL of lysis buffer (25 mM Tris pH 8,15 mM NaCl, 10 mM MgCl_2_, 0.1 mM CaCl_2_, 1 mM phenylmethanesulfonyl fluoride, 0.05 mM dithiothreitol). After treatment with DNase (277 U) for 20 min, the lysates were clarified by centrifuging at 13,000 xg for 15 min and then were incubated with Ezview Red Anti-HA affinity gel (Sigma) for 1 hr at 4°C. The gel was washed 3 times with assay buffer (50 mM Tris, pH 8, 137 mM NaCl, 2.7 mM KCl, 1 mM MgCl_2_) and resuspended in the same buffer to yield a total volume of 60 μL.

Assays were performed in a 96 well plate with the HDAC Fluorometric Assay/Drug Discovery Kit (Enzo Life Sciences). Each 50 μL assay contained 1 μM Trichostatin A (type I and II HDAC inhibitor), 20 μL of the gel-lysate slurry, 200 μM nicotinamide adenine dinucleotide (NAD^+^) and 500 μM *Fluor de Lys*^® ^substrate in assay buffer. For the blank, 1 mM NAM was added before the substrate to inhibit any sirtuin activity. After 1 hr at 37°C, 50 μL of *Fluor de Lys*^® ^developer and 1 mM NAM was added to quench the reaction. The fluorescence intensity of each assay was measured with a Perkin Elmer LS-55, Molecular Devices SpectraMax Gemini EM using an excitation of 355 nm and an emission of 460 nm.

### Plasmid construction

*pIGF-GTW::THD14 for GFP-Thd14 expression*. The *THD14 *gene was amplified from *Tetrahymena *genomic DNA by polymerase chain reaction (PCR) using forward primer 5'-CACCATGAGTTCTGAAATTAGTAAAAC-3' and reverse primer 5'-TCAAAGGTTTTATTTCTTCTCTA-3'. The resulting PCR product was directionally cloned into plasmid pENTR/D-TOPO (Invitrogen) to make plasmid pENTR-*THD14*, and transformed into chemically competent TOP10 *Escherichia coli *cells (Invitrogen). After verification of the cloned *THD14 *sequence, the gene was exchanged with the Gateway cassette in pIGF-GTW [54] by combining 150 ng of pENTR-*THD14 *entry clone with 400 ng of pIGF-GTW and recombinase (LR Clonase II, Invitrogen) and incubating for 20 hrs at 22°C. Following proteinase K digestion, recombination reactions were electroporated into DH10B *Escherichia coli *made electrocompetent by a published method [55]. The resulting pIGF-GTW::*THD14 *plasmid contained the sequence construct to express a GFP fusion with the amino terminus of Thd14 under control of the *MTT1 *promoter. The construct was confirmed by sequencing.

To make the plasmid for expression of HA-Thd14 fusion protein (*pBM2HA::THD14*), the *THD14 *sequence with 630 bp upstream and 532 bp downstream of the gene was cloned into pCR-BluntII-TOPO vector (Invitrogen). *Sal*I and *Avr*II restriction sites were introduced through site-directed mutagenesis (QuikChange, Stratagene) at the start codon and directly after the stop codon respectively (pTHD14SA). After digesting the plasmid with *Sal*I and *Avr*II, the *THD14 *coding sequence was removed and then inserted into pBM2HA-YFG (vector containing the *BTU1 *flanking region surrounding the *MTT1 *promoter upstream of the multiple cloning sites and a sequence encoding a double HA peptide tag) for integration into the *BTU1 *locus of *btu1-1 *cells. The resulting plasmid was named "*pBM2HA-THD14*".

### *Tetrahymena *transformation

The HA-*THD14 *fusion construct (from plasmid *pBM2HA::THD14 *digested with *Kpn*I and *Sac*I) was integrated into the taxol-sensitive beta-tubulin (*btu1-1*) locus of starved *Tetrahymena *cells (strain CU727) through biolistic bombardment as previously described [56]. Transformants were selected by growth in the presence of 20 μM paclitaxel (MP Biomedicals) after 5 hrs of incubation at 30°C without drug. The construct for GFP tagging of *THD14 *(*pIGF-GTW::THD14*) was transformed into strain CU428 by electroporation according to a previously published method [57]. Transformants were selected by growth in the presence of 100 μg/mL paromomycin.

### Reverse-transcriptase PCR, quantitative PCR, and coding sequence determination

Genomic DNA was isolated as described previously (61). Total RNA was isolated from vegetatively dividing, starved, and conjugating cells using the RNeasy Total RNA kit (Qiagen). The cDNA was made as previously described [56] using 2 μg of total RNA for each reaction. The cDNA was used to perform qPCR using SsoFast EvaGreen supermix with low ROX (Bio-Rad) following the manufacturer's directions in a MJ MiniOpticon system (Bio-Rad). Genomic DNA dilutions were used for a standard curve and *CYP1 *or *BTU1 *primers were used for normalization of *THD14 *expression levels. Expression values were set relative to the CU428 starved value in the conjugation expression experiment and logarithmically growing cells in the starvation expression experiment. The following primer sets were used for qPCR; *THD14 *and *CYP1 *primers were designed to flank an intron in order to differentiate between products from cDNA or possible contaminating genomic DNA template for *THD14*:

THD14-QF (5'-CTGATTTGGTCGTCATGG-3')

THD14-QR (5'-ACAGTTCCTTCAGGGTATGTTC-3')

CYP1-QF (5'-AAGGATTAAGGTTAATGTGGTTCA-3')

CYP1-QR (5'-TTTCTGTACTGCAACATAGGGATA-3')

BTU1-QF (5'-GATAGAATCATGGAAACCTTCTC-3')

BTU1-QR (5'-CAAGTGGTTAAGATCACCATAAG-3')

To determine the *THD14 *coding sequence, the entire coding sequence was amplified from cDNA using the primers for construction of pENTR-THD14 (above). The amplified sequence was subcloned onto pENTR plasmid (Invitrogen) and sequenced using M13-forward and M13-reverse primers. The coding sequence was submitted to Genbank, (accession number HQ156951).

### Immunoblot analysis

Whole cell protein lysates were generated from 3 × 10^7 ^cells of both CU428 (wild type) and cells expressing HA-Thd14. Cells were lysed in 700 μL of TLB (350 mM NaCl, 40 mM HEPES pH 7.5, 1% Triton X-100, 10% glycerol, 1 mM dithiothreitol), vortexed for 1 minute, and clarified by centrifuging at 13,000 xg for 15 minutes. Total protein concentration was determined using Bradford Reagent (Bio-Rad). Lysate (100 μg of total protein) was mixed with sodium dodecyl sulfate (SDS) gel loading buffer (50 mM Tris-HCl pH 6.8, 100 mM dithiothreitol, 2% [wt/vol] SDS, 0.1% bromophenol blue, 10% glycerol) and boiled for 5 min. Samples were loaded (20 μg total protein per sample) and resolved by SDS-polyacrylamide gel electrophoresis (PAGE) on a 10% polyacrylamide gel, transferred to nitrocellulose membrane, and incubated first with α-HA antiserum (Covance) diluted 1:2,000 with 5% milk in Tris Buffered Saline (TBS). The membrane was washed 3 × 5 minutes in TBS and then incubated with horseradish peroxidase-conjugated goat α-mouse antiserum (Pierce) diluted 1:10,000 with 5% milk in TBS and developed using SuperSignal West Dura Chemiluminescence Kit (Pierce) and exposed to X-ray film.

### Microscopy

For all live-cell imaging, 1 mL of culture was centrifuged at 2,000 xg and the pellet was incubated for 10 min with 0.1 μg of 4',6 diamino-2-phenylindole dihydrochloride (DAPI; Sigma Chemicals). After dropping 5 μL of 2% methylcellulose on a microscope slide, 1 μL of the pellet was then added and covered with a #1.5 micro cover slip (VWR). For mitochondrial imaging, cell cultures were incubated with 0.5 μg/mL Mito Tracker^© ^Red CMXRos (Invitrogen) for at least 15 hrs and washed 3× with 10 mM Tris Buffer (pH 7.5) prior to sample preparation. Imaging involving mitochondria was performed on an Olympus 1 × 81 fluorescence microscope with a magnification of 100×. All other fluorescence imaging was performed on a Nikon Eclipse E400 fluorescence microscope with a magnification of 40× or 100×. For differential interference contrast (DIC) microscopy, cells were prepared in the same way as for fluorescence microscopy.

### Indirect immunofluorescence and DAPI staining of cells

Growing and conjugating cells were fixed in 3% paraformaldehyde and processed for immunofluorescence microscopy as previously described [58]. For detection of micronuclear linker histone (MLH), cells were incubated with α-MLH antiserum (1:500; generous gift from David Allis). For detection of Nop52, cells were incubated with α-Nop52 antiserum (1:20,000; generous gift from Ronald Pearlman). All primary antisera were detected with rhodamine-conjugated goat anti-rabbit immunoglobulin G (1:100; Jackson ImmunoResearch catalog no. 111-025-003). Fixed cells were counterstained by incubation with 0.1 μg/ml DAPI in 0.1% bovine serum albumin-phosphate-buffered saline for 10 min.

### TUNEL assay

Conjugating cells (CU428 × CU427) were fixed 10 hrs after mixing in 2% paraformaldehyde and stored in 70% ethanol at -80°C for at least 24 hrs. The TUNEL (terminal deoxynucleotidyl transferase dUTP nick end labeling) assay was performed following the manufacturer's instructions for the APO-DIRECT kit (BD Pharmigen). In short, fixed cells were incubated overnight at 30°C in DNA labeling solution (containing FITC dUTP and TdT enzyme) and then exposed for 30 min at room temperature to Propidium Iodide/RNase staining buffer prior to imaging.

## Abbrevations

GFP: green fluorescent protein; HDAC: histone deacetylase; NAM: nicotinamide; NAD^+^: nicotinamide adenine dinucleotide; MLH: micronuclear linker histone; MTT1: metallothioneine; OM: old macronucleus; PCR: polymerase chain reaction; TBS: Tris buffered saline; Thd14: *Tetrahymena *histone deacetylase 14; TUNEL: terminal deoxynucleotidyl transferase dUTP nick end labeling

## Competing interests

The authors declare that they have no competing interests.

## Authors' contributions

KMS performed the TUNEL assays, NAM experiments, HDAC assay, GFP-Thd14/mitotracker microscopy, and helped draft and revise the manuscript. SF produced the genetic constructs and transformed cell lines, initial gene expression profiles, GFP-Thd14 nuclear localization and Nop52 immunofluorescence results, and performed some of the bioinformatics analyses. KAC performed the qRT-PCR, some bioinformatics analyses, all statistics, and helped revise the manuscript. JJS assisted with transformations, helped draft and revise the manuscript, and assisted with supervision of experiments. EAW conceived the idea, drafted the manuscript, supervised the experiments, and performed some of the imaging. All authors read and approved the final manuscript.

## Supplementary Material

Additional file 1 **"Time dependence of nicotinamide treatment effects"**. This data illustrates a decrease in the old macronucleus retention phenotype with NAM treatment later in conjugation. Click here for file

Additional file 2 **"Thd14 sirtuin core domain resembles that of other class I sirtuins"**. This is an alignment of Thd14 with the sirtuin domains of yeast and human enzymes. It shows strong conservation of active site residues and metal binding residues. Click here for file

Additional file 3 **"Thd14 has histone deacetylase activity"**. This is data from an experiment testing for histone deacetylase activity. It shows that Thd14 deacetylates histone amino-terminal peptides. Click here for file
