# Supplementary material for: Sirtuin-mediated nuclear differentiation and programmed degradation in Tetrahymena
Source: BMC Cell Biol. 2011 Sep 21;12:40. doi: 10.1186/1471-2121-12-40 (PMC3191509; doi:10.1186/1471-2121-12-40)
Supplement: Additional file 1 — "Time dependence of nicotinamide treatment effects". This data illustrates a decrease in the old macronucleus retention phenotype with NAM treatment later in conjugation. [file 1471-2121-12-40-S1.DOC]

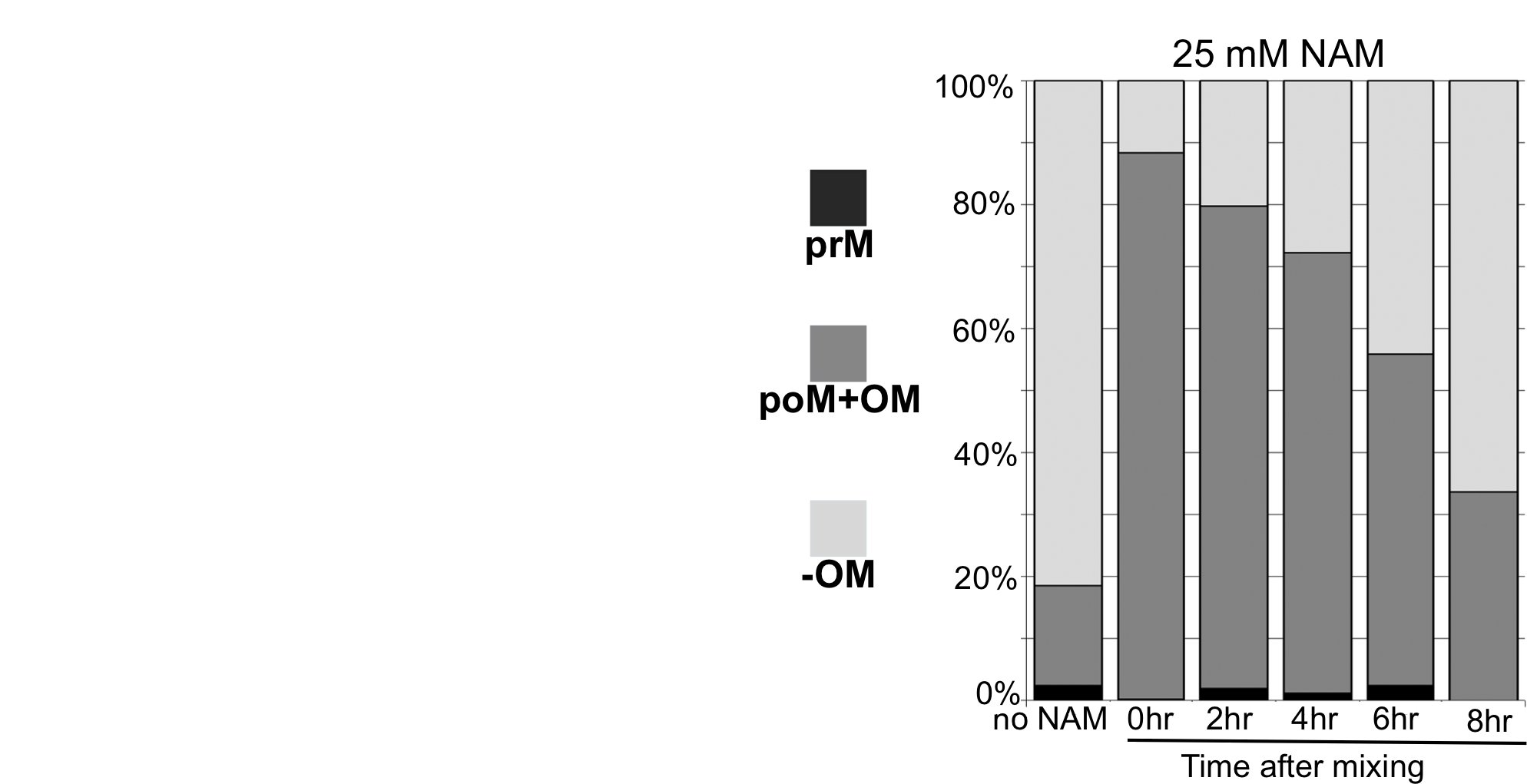


**Additional File 1.** **Time dependence of** **nicotinamide treatment**. 25 mM nicotinamide was added to conjugating cells at the various times indicated below each bar on the x-axis and analyzed at 24 hrs post-mixing. The percentage of conjugating pairs at each of the three stages is illustrated in Figure 1a by brackets: “PrM” are cells in pre-meiosis (black); “pOM+OM” are post-meiotic stages that still have a parental macronucleus (old macronucleus; OM), “-OM” are cells that lack an old macronucleus post-degradation (light gray).
