# Supplementary material for: Sirtuin-mediated nuclear differentiation and programmed degradation in Tetrahymena
Source: BMC Cell Biol. 2011 Sep 21;12:40. doi: 10.1186/1471-2121-12-40 (PMC3191509; doi:10.1186/1471-2121-12-40)
Supplement: Additional file 2 — "Thd14 sirtuin core domain resembles that of other class I sirtuins". This is an alignment of Thd14 with the sirtuin domains of yeast and human enzymes. It shows strong conservation of active site residues and metal binding residues. [file 1471-2121-12-40-S2.DOC]

Hst1 DHFTAT--------LR--NAKKILVLTGAGVSTSLGIPDFRSS-EGFYSK

Hst2 RKIAAH--------MKSNPNAKVIFMVGAGISTSCGIPDFRSPGTGLYHN

SIRT1 EDAVKL--------LQ--ECKKIIVLTGAGVSVSCGIPDFRSR-DGIYAR

SIRT2 EGVARY--------MQSERCRRVICLVGAGISTSAGIPDFRSPSTGLYDN

SIRT3 -------------------------MVGAGISTPSGIPDFRSPGSGLYSN

Sir2 DHFIQK--------LH--TARKILVLTGAGVSTSLGIPDFRSS-EGFYSK

Thd14 RDISPDQEKNQDDKYNNRQFNRIVFLTGAGISVSAGIPDFRTPGSGLYSQ

:.***:*.. ******: *:* .

Hst1 IR--HLGLEDPQDVFNLDIFLQDPSVFYNIAHMVLPPENMYSPLHSFIKM

Hst2 LA--RLKLPYPEAVFDVDFFQSDPLPFYTLAKELYPGNFRPSKFHYLLKL

SIRT1 LAVDFPDLPDPQAMFDIEYFRKDPRPFFKFAKEIYPGQFQPSLCHKFIAL

SIRT2 LE--KYHLPYPEAIFEISYFKKHPEPFFALAKELYPGQFKPTICHYFMRL

SIRT3 LQ--QYDLPYPEAIFELPFFFHNPKPFFTLAKELYPGNYKPNVTHYFLRL

Sir2 IK--HLGLDDPQDVFNYNIFMHDPSVFYNIANMVLPPEKIYSPLHSFIKM

Thd14 LQ--KYKLPYPEAIFEINYFKHHPQPFYTLCKEFSSCGSHFTSSHFFIAE

: * *: :*: * .* *: :.: . . . * ::

Hst1 LQDKGKLLRNYTQNIDNLESYAGIDPDKLVQCHGSFATASCV--TCHWQI

Hst2 FQDKDVLKRVYTQNIDTLERQAGVKDDLIIEAHGSFAHCHCI--GCGKVY

SIRT1 SDKEGKLLRNYTQNIDTLEQVAGIQ--RIIQCHGSFATASCL--ICKYKV

SIRT2 LKDKGLLLRCYTQNIDTLERIAGLEQEDLVEAHGTFYTSHCVSASCRHEY

SIRT3 LHDKGLLLRLYTQNIDGLERVSGIPASKLVEAHGTFASATCT--VCQRPF

Sir2 LQMKGKLLRNYTQNIDNLESYAGISTDKLVQCHGSFATATCV--TCHWNL

Thd14 TNRRNRLLINFSQNIDGLELEAGLPESKLVQAHGHFRTAKCV--NCKKVA

. .. * ::**** ** :*: :::.** * . * *

Hst1 PGEKIFENIRNLEL---PLCPYCYQKRKQYFPMSNGNNT----VQTNINF

Hst2 PPQVFKSKLAEHPIKDFVKCDVCG--------------------------

SIRT1 DCEAVRGDIFNQVV---PRCPRCPAD------------------------

SIRT2 PLSWMKEKIFSEVT---PKCEDCQ--------------------------

SIRT3 PGEDIRADVMADRV---PRCPVCT--------------------------

Sir2 PGERIFNKIRNLEL---PLCPYCYKKRREYFPEGYNNKVGVAASQGSMSE

Thd14 DIELFNEAVKNDKI---CYCKECE--------------------------

. . : * *

Hst1 NSP-ILKSYGVLKPDMTFFGEALPSRFHKTIRKDI-------------LE

Hst2 ---------ELVKPAIVFFGEDLPDSFSETWLNDSEWLREKITTSGKHPQ

SIRT1 ------EPLAIMKPEIVFFGENLPEQFHRAMKYDK-------------DE

SIRT2 ---------SLVKPDIVFFGESLPARFFSCMQSDF-------------LK

SIRT3 ---------GVVKPDIVFFGEPLPQRFLLHV-VDF-------------PM

Sir2 RPPYILNSYGVLKPDITFFGEALPNKFHKSIREDI-------------LE

Thd14 --------EGIVKPDIVFFGESLPQSFFQQI-DSL-------------NK

::** :.**** ** * .

Hst1 CDLLICIGTSLKVAPVSEIVNMVPSHVPQILINRDMVTH-----------

Hst2 QPLVIVVGTSLAVYPFASLPEEIPRKVKRVLCNLETVGDFK---------

SIRT1 VDLLIVIGSSLKVRPVALIPSSIPHEVPQILINREPLPH-----------

SIRT2 VDLLLVMGTSLQVQPFASLISKAPLSTPRLLINKEKAGQSDPFLGMIMGL

SIRT3 ADLLLILGTSLEVEPFASLTEAVRSSVPRLLINRDLVGPLA---------

Sir2 CDLLICIGTSLKVAPVSEIVNMVPSHVPQVLINRDPVKH-----------

Thd14 ADLVFVMGTSLKVFPFAALVDLFKEDVPIVLINRENPGI-----------

*:: :*:** * *.: : . . :* * :

Hst1 ----------AEFDLNLLGFCDDVASLVAKKCHWDIP

Hst2 -------ANKRPTDLIVHQYSDEFAEQLVEELGWQED

SIRT1 ----------LHFDVELLGDCDVIINELCHRLGGEYA

SIRT2 GGGMDFDSKKAYRDVAWLGECDQGCLALAELLGWKKE

SIRT3 -------WHPRSRDVAQLGDVVHGVESLVELLGWTEE

Sir2 ----------AEFDLSLLGYCDDIAAMVAQKCGWTIP

Thd14 ---------KRRRFLFLEGEIDDNVEKIMKDISWDFP

: : .

**Additional File 2: Thd14 sirtuin core domain resembles that of other class I sirtuins.** The sirtuin core domain of the class I sirtuins Sir2, Hst1 through 4 and SIRT1 through 3 and Thd14 was aligned using TCOFFEE. Chemically similar residues are indicated by gray shading while identical residues are indicated by black shading. Yellow highlighted residues indicate the sirtuin core domain active site while green highlighting indicates metal binding sites as determine by ExPASy – PROSITE. Thd14 possess the same active site and metal binding residues as the other class I sirtuins.
