# Supplementary material for: Sirtuin-mediated nuclear differentiation and programmed degradation in Tetrahymena
Source: BMC Cell Biol. 2011 Sep 21;12:40. doi: 10.1186/1471-2121-12-40 (PMC3191509; doi:10.1186/1471-2121-12-40)
Supplement: Additional file 3 — "Thd14 has histone deacetylase activity". This is data from an experiment testing for histone deacetylase activity. It shows that Thd14 deacetylates histone amino-terminal peptides. [file 1471-2121-12-40-S3.DOC]

**
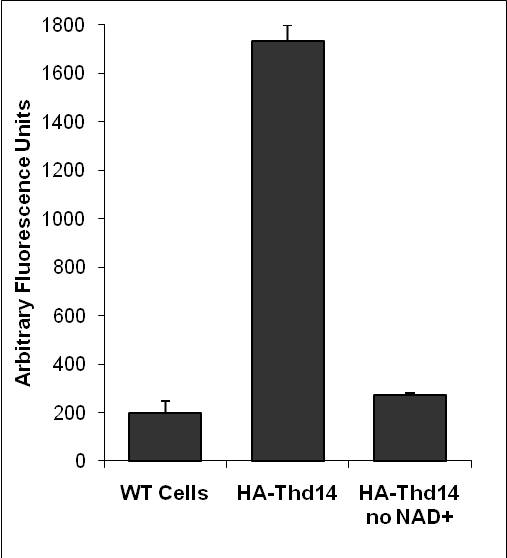
**

**Additional File 3. Thd14 deacetylates the amino termini of histones**. Lysate from cells expressing untagged Thd14 (wt cells) or Thd14 fused with HA (HA-Thd14) were incubated with anti-HA affinity beads. After washing, the beads were incubated with Trichostatin A (type I and II HDAC inhibitor), nicotinamide adenine dinucleotide (NAD^+^) and *Fluor de Lys*® substrate (N-terminal peptides) in assay buffer. After 1 hr at 37°C, *Fluor de Lys*® developer and 1 mM NAM was added to quench the reaction and fluorescence intensities were collected. (λ_ex_ =355 nm; λ_em_ = 460 nm). For the blank, 1 mM NAM was added before the substrate to inhibit any sirtuin activity.
